# Preparation of Reusable Porous Carbon Nanofibers from Oxidized Coal Liquefaction Residue for Efficient Adsorption in Water Treatment

**DOI:** 10.3390/ma16103614

**Published:** 2023-05-09

**Authors:** Yaoyao Chen, Kefu Wang, Liqin Cao, Xueli Huang, Yizhao Li

**Affiliations:** 1 State Key Laboratory of Chemistry and Utilization of Carbon Based Energy Resources, College of Chemical Engineering and Technology, Xinjiang University, Urumqi 830017, China; 2 Yangtze Delta Region Institute (Huzhou), University of Electronic Science and Technology of China, Huzhou 313001, China

**Keywords:** coal liquefaction residue, electrostatic spinning, porous carbon nanofibers, organic dyes, adsorption

## Abstract

Porous carbon nanofibers are commonly used for adsorption processes owing to their high specific surface area and rich pore structure. However, the poor mechanical properties of polyacrylonitrile (PAN)-based porous carbon nanofibers have limited their applications. Herein, we introduced solid waste-derived oxidized coal liquefaction residue (OCLR) into PAN-based nanofibers to obtain activated reinforced porous carbon nanofibers (ARCNF) with enhanced mechanical properties and regeneration for efficient adsorption of organic dyes in wastewater. This study examined the effects of contact time, concentration, temperature, pH, and salinity on the adsorption capacity. The adsorption processes of the dyes in ARCNF are appropriately described by the pseudo-second-order kinetic model. The maximum adsorption capacity for malachite green (MG) on ARCNF is 2712.84 mg g^−1^ according to the fitted parameters of the Langmuir model. Adsorption thermodynamics indicated that the adsorptions of the five dyes are spontaneous and endothermic processes. In addition, ARCNF have good regenerative performance, and the adsorption capacity of MG is still higher than 76% after 5 adsorption-desorption cycles. Our prepared ARCNF can efficiently adsorb organic dyes in wastewater, reducing the pollution to the environment and providing a new idea for solid waste recycling and water treatment.

## 1. Introduction

Dyes are widely used in a variety of industries including textiles, apparel, printing and cosmetics [1,2,3]. Synthetic dyes with a high output and low cost have arisen in response to the demands of human life, which have steadily gained acceptance and become widely used [4,5,6]. However, the direct discharge of wastewater contaminated by dyes has caused serious environmental problems [7]. Most dyes with complex aromatic structures are long-lasting in the nature and difficult to decompose, which is extremely dangerous for both human health and the environment [8]. Hence, it is urgent to explore efficient methods to treat wastewater containing dyes.

The methods commonly employed for dye wastewater treatment are chemical methods, such as electrochemical oxidation [9] and photocatalytic degradation [10], and physical methods, including adsorption [11,12,13] and membrane-filtration [14]. Among which the adsorption method has the advantages of low cost, high efficiency and flexible operation, making it an affordable and secondary pollution-free technology for dye wastewater treatment [15,16,17]. In recent years, porous carbon materials have attracted increasing attention for their unique structural properties and chemical stability, which are widely used as high-performance dye adsorbents [18,19,20]. For instance, Saghir et al. [21] prepared porous carbon with large specific surface area and rich pore structure from pistachio shells, which was applied for the adsorption of Congo red and methylene blue (MB) in wastewater. Qi et al. [22] developed a double-template method to prepare hierarchical porous carbon for the adsorption of MB and rhodamine B (RB) from aqueous solutions. Wang et al. [23] fabricated porous carbon with good adsorption performances for both cationic and anionic dyes through a two-step thermolysis method. However, recovery of powdered porous adsorbents requires filtration or centrifugation, increasing the cost and process complexity of wastewater treatment [24]. Compared to conventional methods, fiber membrane adsorption is considered the most attractive method for wastewater treatment [25,26]. Porous carbon nanofibers with large specific surface area, high porosity, and rich active sites are convenient to collect and recycle, and exhibit great potential for application in the field of adsorption [15].

As a bulk solid waste, coal liquefaction residue (CLR) has high carbon content and is prone to polymerization as well as cross-linking. Its conversion into high value-added carbon materials is significant [27,28,29]. Recently, some researchers have prepared a variety of carbon fibers using CLR as carbon precursors, providing valuable ideas for the conversion of CLR into functional carbon materials. Zhou et al. [30] synthesized carbon microfibers directly by arc-jet plasma method using CLR as raw material. Qiao et al. [31] prepared polyacrylonitrile-based carbon nanofibers from extracts of CLR by electrospinning technology. Xiao et al. [32] used CLR as raw material to prepare carbon nanofiber/carbon foam composites by template method and chemical vapor deposition method. However, carbon fibers prepared from CLR tend to exhibit poor mechanical properties and are therefore not conducive to be an efficient adsorbent. The conversion of CLR into functionalized carbon nanofibers and their application for adsorption of organic dyes in wastewater is needed further improvement [33].

According to our previous work [34], solid waste-derived oxidized CLR (OCLR) with high conjugation, high crystallinity, and high oxygen content were prepared by liquid-phase oxidation of CLR. Herein, we spun OCLR into polyacrylonitrile (PAN)-based fibers by electrostatic spinning technology to prepare porous carbon nanofibers (ARCNF) with enhanced mechanical properties as well as easy recycling. ARCNF showed excellent adsorption performance as a highly efficient and renewable adsorbent for the adsorption of common cationic (malachite green, MG) and anionic dyes (reactive red 120, RR-120) in wastewater. To verify the universality of the adsorption of ARCNF on cationic dyes, we also performed adsorption on methyl orange (MO), MB and RB. The structural composition of ARCNF was characterized. The effects of adsorption time, dye concentration, solution pH, and salinity on the adsorption performance were investigated, as well as discussing the adsorption thermodynamics and recycling performance. Our work may provide new thought for highly efficient water treatment and high-value-added utilization of solid waste resources.

## 2. Materials and Methods

### 2.1. Materials

OCLR were prepared by liquid-phase oxidation method using CLR (Shenhua Ordos Coal and Oil Branch). The details are given in our previous work [34].

The dyes required for the experiments are shown in Table 1. MO, MB and RR-120 were purchased from Tianjin Fuchen Chemical Reagent Factory (Tianjin, China). MG, RB and potassium hydroxide (KOH) were obtained from Shanghai Aladdin Co., Ltd. (Shanghai, China) Hydrochloric acid (HCl) was purchased from Dicheng Chemical Co., Ltd. (Urumchi, China) Polyacrylonitrile (PAN, J&K Scientific Co., Ltd., Beijing, China, average molecular weight: 150,000 Da), N, N-dimethylformamide (DMF, AR, Tianjin Guangfu Fine Chemical Research Institute, Tianjin, China) and other reagents were used as received without further purification.

### 2.2. Preparation of ARCNF

**Figure 1:**
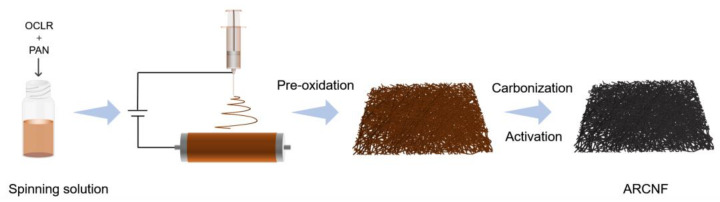
Schematic illustration for the preparation of ARCNF.

The schematic illustration of sample preparation is shown in Figure 1. 0.5 g PAN powder and 0.25 g OCLR were added to 5 mL of DMF solution and mixed uniformly under magnetic stirring and ultrasonication for 36 h. Electrostatic spinning was performed at a positive voltage of 18 kV and a negative voltage of 2 kV. The roller speed was set to 80 r min^−1^ and the needle translation speed was 100 mm min^−1^. The spinning fibers were pre-oxidized in air at 280 °C for 1 h at a rate of 2 °C min^−1^, and then transferred to a tube furnace followed by heating to 500 °C for 1 h at a rate of 2 °C min^−1^ under N_2_ atmosphere. After immersion of the carbonized fibers using KOH solution, the KOH to carbon precursor mass ratio was 1:3, chemical activation was performed by heating to 800 °C for 1 h at a rate of 5 °C min^−1^. The activated fibers were first immersed in 1 M HCl to remove inorganic salts, and then washed repeatedly with deionized water to neutral. The sample was obtained by drying in an oven at 80 °C, named as ARCNF. In comparison, the porous carbon nanofibers prepared by the above steps without adding OCLR was named ACNF.

### 2.3. Characterization of ARCNF

The sample was characterized by scanning electron microscopy (SEM, Hitachi S-4800, Tokyo, Japan), high-resolution transmission electron microscopy (HRTEM, JEOL-2100F, Tokyo, Japan), X-ray diffraction (XRD, Bruker D8 Advance, Mannheim, Germany), Raman (Bruker SENTERRA), and X-ray photoelectron spectroscopy (XPS). The zeta potential of ARCNF in the solid state was directly measured by an Electrokinetic Analyzer (SurPass 3, Anton Paar, Graz, Austria). The surface area was obtained from N_2_ adsorption-desorption isotherms using a volumetric adsorption apparatus (ASAP 2460, Micromeritics, Norcross, GA, USA). The pore size distribution was calculated by Non-Local Density Functional Theory (NLDFT) method. The surface area was obtained by Braunauer-Emmett-Teller (BET) method.

### 2.4. Adsorption of Organic Dyes

The stock solution of MO, RB, RR-120, MG, and MB with a concentration of 400 mg L^−1^ were prepared in deionized water, and then diluted as the stock solution. All adsorption experiments were performed three times for each experiment using 160 rpm min^−1^ thermostatic water bath shaker. The absorbance at the maximum absorption wavelength of the five dye stock solutions was measured by UV-Visible spectrophotometer. The effects of some factors on the adsorption capacity of ARCNF were considered, such as contact time, dyes concentration, temperature, pH, and salinity, respectively. The adsorption capacity Q_t_ (mg g^−1^) of five dyes was calculated by following equation [3]:

(1) $$Q_{t}=\frac{V}{m}\;\left(C_{0}-C_{t}\right)$$

where C_0_ (mg L^−1^) and C_t_ (mg L^−1^) are the initial dyes solution concentration and the concentration of dyes solution with time t (h), V (L) is the volume of dyes solution, and m (g) is the mass of the adsorbent.

### 2.5. Adsorption Experiment

In the kinetic experiments, 1 mg ARCNF was used to adsorb 40 mL of different dyes solution with 10 mg L^−1^ at 298 K for contact time of 1 h, 2 h, 4 h, 8 h,12 h, 24 h, 36 h, 48 h, 60 h, 72 h, 84 h, respectively. The dyes concentrations in the filtrate were measured using a UV-Visible spectrophotometer.

In the isotherm experiments, 1 mg ARCNF was used to adsorb 40 mL of different dyes solution with 10, 20, 30, 40, 50, 60, 70 mg L^−1^, respectively. The adsorption capacity of dyes at 298 K for 72 h was determined.

In the thermodynamic experiments, 1 mg ARCNF was used to adsorb 40 mL of different dyes solution with 10, 20, 30, 40, 50 mg L^−1^ at 288 K, 298 K, and 308 K, respectively. The adsorption capacity with contact time for 72 h was measured.

Effect of pH: the pH of the adsorption solution was adjusted to 3, 5, 7, 9 and 11 with 0.1 M HCl and 0.1 M NaOH, respectively. 1 mg ARCNF was used to adsorb 40 mL of solution with 20 mg L^−1^ at different pH. The adsorption capacity of dyes at 298 K for 72 h was evaluated.

Effect of salinity: 1 mg ARCNF was used to adsorb 40 mL of solution with different salt concentration (NaCl) of 10, 20, 30, 40, and 50‰, respectively. The adsorption capacity of dyes at 298 K for 72 h was measured.

## 3. Results and Discussion

### 3.1. Structure and Morphologies

**Figure 2:**
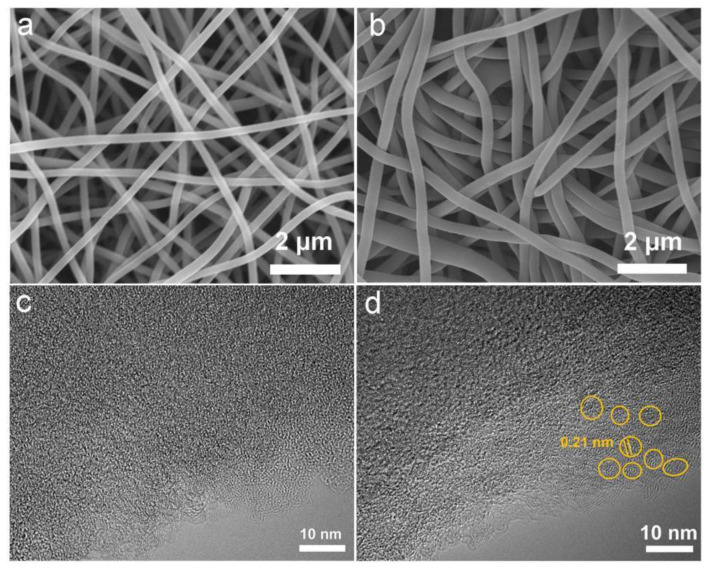
SEM images (**a**) ACNF and (**b**) ARCNF. HRTEM images of (**c**) ACNF and (**d**) ARCNF.

**Figure 3:**
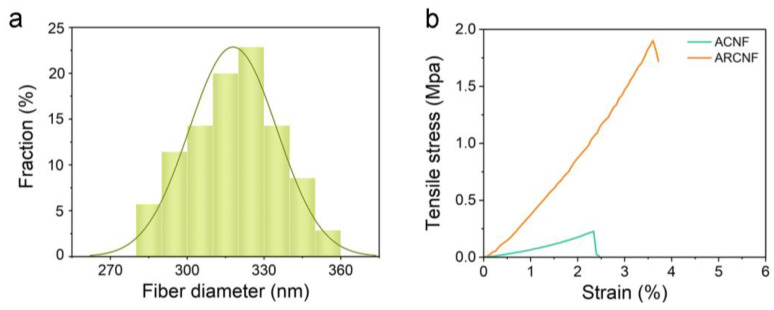
(**a**) Diameter distribution of ARCNF. (**b**) The stress-strain curves of ACNF and ARCNF.

Figure 2a,b show the SEM images of ACNF and ARCNF, respectively. Both samples exhibit carbon nanofibers with random arrangement and uniform diameters. The average diameter of the nanofibers increases from 220 nm (Figure S1) in ACNF to 320 nm (Figure 3a) in ARCNF after adding OCLR, which is owing to the increased concentration of spinning solution [35]. From the HRTEM images in Figure 2c,d, ACNF shows a large number of amorphous regions. ARCNF presents a uniformly distributed crystalline structure at the edge with a lattice parameter of 0.21 nm (See the yellow circles in Figure 2d), which corresponds to the crystalline plane of OCLR (Figure S2), indicating that the addition of OCLR can significantly improve the crystallinity of the nanofibers [36].

**Figure 4:**
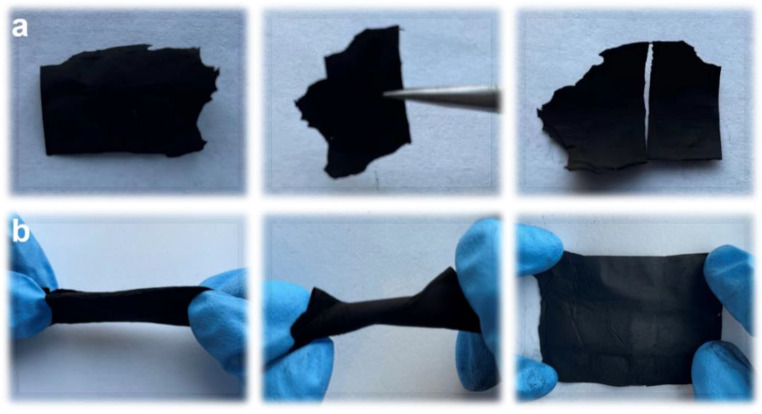
Photographs of (**a**) ACNF and (**b**) ARCNF after folding/twisting and releasing.

Young’s modulus is used to measure the mechanical properties of materials based on the ratio of tensile stress to tensile strain during the stretching process [37]. From Figure 3b, the Young’s modulus of the fiber increases from 9.7 MPa in ACNF to 52.8 MPa in ARCNF, indicating the formation of an enhanced phase within the ARCNF [38]. During the carbonization process, the oxygen-containing functional groups at the edges of ARCNF are reacted with the groups on the polyacrylonitrile chains to form a strong force. Even after chemical activation, the strong and dense carbon skeleton is retained inside the porous carbon nanofibers, improving its mechanical properties. To further investigate the flexibility of the samples, we folded and twisted them, and then observed the integrity of the samples after release. In Figure 4, ACNF is easy to break after folding due to the inherent cracks and pores, which shows poor mechanical strength. ARCNF with OCLR can recover its initial shape after folding and twisting, which demonstrates that the addition of OCLR enhances mechanical strength.

**Figure 5:**
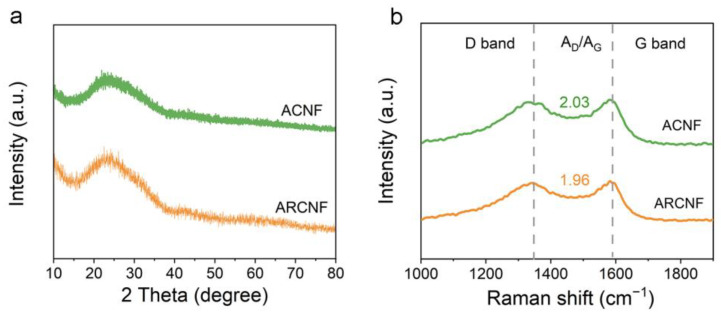
(**a**) XRD patterns, (**b**) Raman spectra of ACNF and ARCNF.

To investigate the crystallinity and defect density of ACNF and ARCNF, XRD and Raman analysis were performed. As can be seen from Figure 5a, the samples all show broad and sharp diffraction peaks around 24°, corresponding to the (002) crystal plane of carbon [39]. Notably, the ARCNF exhibits a stronger (002) diffraction peak than the ACNF, indicating an enhanced of the crystallinity, which is consistent with the HRTEM image. The Raman spectra (Figure 5b) shows two distinct diffraction peaks at 1350 and 1590 cm^−1^ for both samples, belonging to the D band and G band of carbon, respectively [40]. The A_D_/A_G_ value decreases from 2.03 in ACNF to 1.96 in ARCNF, which suggests that the addition of highly crystalline OCLR after heat treatment can reduce the internal defect density of the carbon nanofibers and reveals a higher crystallinity.

**Figure 6:**
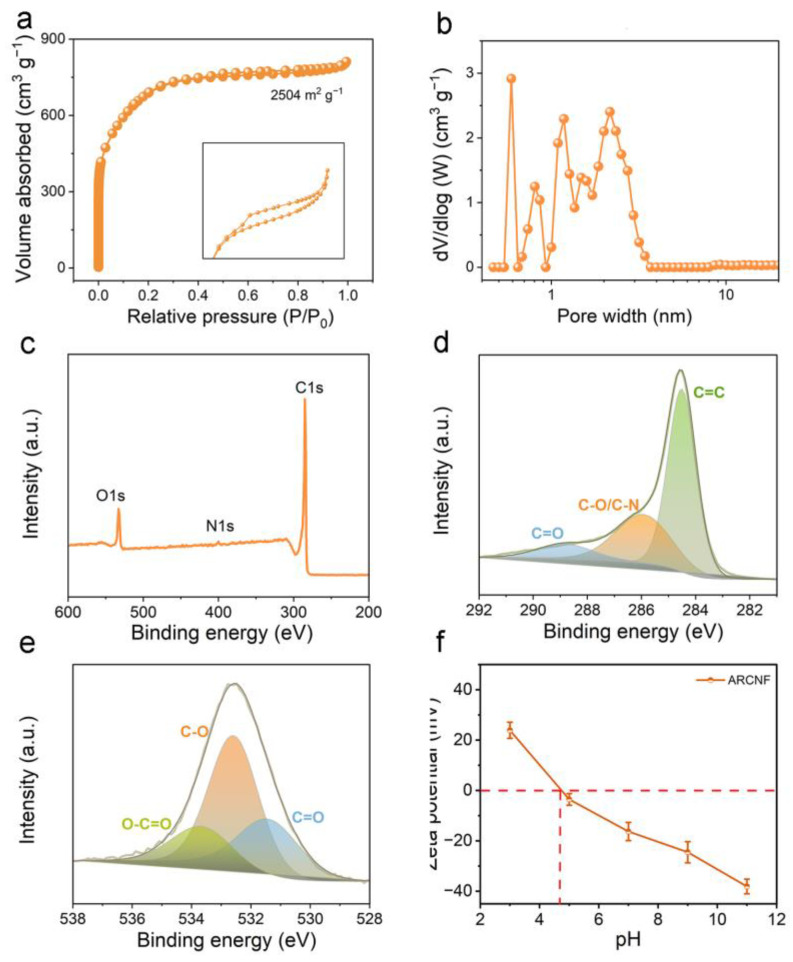
(**a**) N_2_ adsorption–desorption isotherm, (**b**) pore size distribution, (**c**) XPS survey spectra, (**d**) XPS C 1s spectra, (**e**) XPS O 1s spectra, and (**f**) zeta potential of ACRNF.

The large specific surface area and rich pore structure can effectively promote the adsorption toward organic pollutants. The specific surface area and pore structure of ARCNF were characterized by N_2_ adsorption and desorption curves [41]. From Figure 6a, ARCNF exhibits both type I and type IV isotherms. In the low-pressure region (P/P_0_ < 0.1), the adsorption curves exhibit a rapid increasing trend, indicating the presence of a microporous structure [12]. In the medium-pressure region (0.45 < P/P_0_ < 0.9), the appearance of a hysteresis loop can be observed, suggesting a certain mesopore [42]. As can be seen from Figure 6b, the micropores of ARCNF are mainly dominated by 0.59 to 1.18 nm, and the mesopores are distributed at about 2.51 nm. The abundant micropores provide more adsorption sites for dyes, while the mesopores also provide transport channels for ions [43]. In addition, ARCNF has a large specific surface area of 2504 m^2^ g^−1^ and a high total pore volume of 1.25 cm^3^ g^−1^ (Table S1), which is helpful for the efficient adsorption of organic dyes.

The elemental composition and chemical state of the samples were characterized by XPS [41]. Figure 6c illustrates the XPS survey spectra of ARCNF. The diffraction peaks of C 1s (88.1 at%), O 1s (9.7 at%), and N 1s (2.2 at%) can be seen at 284.6 eV, 532.6 eV, and 400.0 eV, respectively. The high-resolution C 1s spectrum can be deconvolved into three peaks of C=C (284.6 eV), C−O/C−N (285.9 eV), and C=O (288.9 eV) components, respectively (Figure 6d and Table S2) [44]. The high-resolution O 1 s spectrum can be classified into three peaks at 531.2 eV, 532.6 eV, and 533.8 eV, which attributed to C=O, C−O, and O−C=O components, respectively (Figure 6e). It can be seen that the ARCNF is rich in oxygen functional group sites, which contribute to the adsorption of dyes [45].

Figure 6f shows the zeta potential plot of ARCNF. The point of zero potential (pH_zpc_) of ARCNF is 4.78. ARCNF has a positive surface charge at pH < 4.78, which can adsorb anions under the action of coulomb forces. When pH = 4.78, the surface charge of the adsorbent is zero. At pH > 4.78, the surface of ARCNF is negatively charged and will adsorb cations. Therefore, the electrostatic effect promotes the adsorption of ARCNF in cationic dyes.

### 3.2. Adsorption of Organic Dyes

#### 3.2.1. Adsorption Kinetic of Organic Dyes on ARCNF

**Figure 7:**
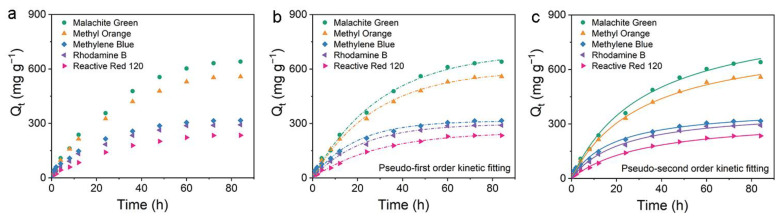
Adsorption kinetics of different organic dyes on ARCNF: (**a**) contact time, (**b**) pseudo-first order kinetic fitting, (**c**) pseudo-second order kinetic fitting.

Due to the poor mechanical properties of ACNF, it is difficult to collect, so we performed a series of adsorption experiments on ARCNF. Firstly, the kinetics of organic dyes adsorption by ARCNF was investigated. Contact time is an essential factor affecting dye adsorption, and the effect of contact time on the adsorption of five dyes in ARCNF is shown in Figure 7a [21]. It can be seen that the adsorption of the five dyes, MG, MO, MB, RB, and RR-120, exhibits two phases: a very fast initial adsorption for the first 24 h, followed by a longer slow adsorption, and the dynamic adsorption equilibrium was basically reached at 72 h. The optimal contact time for the five dyes with ARCNF is 72 h.

Kinetic models are important to evaluate the adsorption kinetics and to examine the adsorption efficiency [43]. The pseudo-first-order kinetic model (PFOKM) and pseudo-second-order kinetic model (PSOKM) were used to describe the sorption kinetics, which are expressed as following equations [46]:

(2) $${\mathrm{PFOKM}:\;Q}_{t}{=Q}_{e}\left({1-\;e}^{{-k}_{1}t}\right)$$

(3) $${\mathrm{PSOKM}:\;Q}_{t}=\frac{Q_{e}{}^{2}k_{2}t}{{1+Q}_{e}k_{2}t}$$

where Q_t_ (mg g^−1^) is the amount of absorption at time t (h), Q_e_ (mg g^−1^) represents the amount of absorption at equilibrium, k_1_ (h^−1^) and k_2_ (mg^−1^ g^−1^ h^−1^) are the adsorption rate constants for PFOKM and PSOKM, and *t* is adsorption time (h), respectively.

The adsorption kinetic models of ARCNF for MO, RB, RR-120, MG and MB dyes are shown in Figure 7b,c. The parameters and correlation coefficients (R^2^) of the PFOKM and PSOKM fits are shown in Table 2. It shows that the values of R^2^ for the PSOKM (0.992–0.998) are all higher than that for the PFOKM (0.987–0.997), and the chi-square error values (X^2^) are all smaller than that of PFOKM, indicating that the PSOKM is more suitable for the dynamic adsorption processes of the five dyes [47]. To verify the accuracy of the experimental results, the confidence intervals of PSOKM for different dyes were analyzed (Figure S3). The fitted results show that the confidence intervals of the adsorption kinetic curves for each dye are within 95%, indicating their accuracy and credibility. The PSOKM confirms that its rate-limiting step is chemisorption, involving the action of valence forces or the exchange of electrons between the dyes and the adsorbents. In general, the adsorption of dyes is controlled by the liquid-phase mass transfer rate or the intra-particle mass transfer rate [48]. The adsorbent component may be introduced from the solution into the solid phase by an intra-particle diffusion process, which is the rate-limiting step in many adsorption processes [43].

#### 3.2.2. Adsorption Isotherm of Organic Dyes on ARCNF

The adsorption isotherm can reflect the equilibrium state of the adsorbate combined with the adsorbent in solution, further illustrating the adsorption mechanism of ARCNF [7]. The effects of different initial quality concentrations of the five dyes on the adsorption performance at a contact time of 72 h are shown in Figure S4. The results indicate that the adsorption capacity increases gradually with increasing dye concentration (10–70 mg L^−1^), but when the adsorption sites in the material are fully occupied, the adsorption capacity no longer increases to reach adsorption equilibrium [39].

Langmuir and Freundlich isotherm models were used to fit the adsorption isotherm data as shown in Table 3. The isotherm equations are expressed as follows [45]:

(4) $${\mathrm{Langmuir}\;\mathrm{model}:\;Q}_{e}=\frac{Q_{m}K_{L}c_{e}}{{1+K}_{L}c_{e}}$$

(5) $${\mathrm{Freundlich}\;\mathrm{model}:\;Q}_{e}{=K}_{F}c_{e}{}^{1/n}$$

where Q_m_ (mg g^−1^) is the maximum adsorption capacity of per gram of ARCNF, C_e_ (mg L^−1^) is the equilibrium concentration of organic dyes, K_L_ (L g^−1^) and K_F_ (L g^−1^) are the affinity constants of the Langmuir and Freundlich model, respectively, and n represents the surface heterogeneity factor.

From the results of fitting the adsorption isotherms of the five dyes (Table 3), it can be seen that the Langmuir isotherm model has the highest correlation coefficients (R^2^ > 0.975) and the lowest X^2^ compared to the Freundlich model. Therefore, the Langmuir isotherm adsorption model can be used to describe the adsorption process of five organic dye, indicating that the adsorption mechanism of them in ARCNF is mainly based on single-layer adsorption. The active sites on the surface of the adsorbent are homogeneously distributed [26]. In addition, the K_L_ values ranged from 0.010 to 0.030 are very close to zero, indicating that the adsorption is effective, and there is a robust affinity between the dyes and ARCNFs [47]. From the Langmuir model, the maximum adsorption capacity of ARCNF for MG, MO, MB, RB, and RR-120 dye is 2712.84, 2210.58, 1017.27, 867.21, and 719.49 mg g^−1^, respectively, indicating that the ARCNF has sufficient adsorption capacity for all the five organic dyes. Table 4 records the comparison of the maximum adsorption capacity of different dyes by various adsorbent materials stated in the literature. It indicates that the adsorption of ARCNF on common dyes is relatively higher than other adsorbents. In our work, the high value-added carbon material (ARCNF) obtained by using solid waste CLR has a great advantage in the adsorption of organic dyes.

#### 3.2.3. Adsorption Thermodynamics of Organic Dyes on ARCNF

The adsorption thermodynamics of the five dyes on ARCNF was further investigated to explore the adsorption mechanism. The thermodynamic parameters were calculated using a Van’t Hoff plot, as shown in the following equations [9]:

(6) $$G^{0}=-\mathrm{RT}\cdot \ln\left(\frac{q_{e}}{C_{e}}\right)$$

(7) $${\ln(q}_{e}{/C}_{e}{)=-\Delta H}^{0}{/\mathrm{RT}+\Delta S}^{0}/R$$

where q_e_ and C_e_ are adsorption capacity and concentration at adsorption equilibrium, ΔG (kJ mol^−1^) represents the free energy change, R (8.314 J K^−1^ mol^−1^) represents the universal gas constant, T (K) represents the system temperature, ΔH (kJ mol^−1^) is the enthalpy change, and ΔS (J K^−1^ mol^−1^) is the entropy change.

From the Figures S5 and S6, the adsorption capacity increases with increasing temperature, indicating that higher temperature is favorable for the adsorption of the five dyes. Therefore, the adsorption of the five dyes is essentially an endothermic process [41]. This may be because of the decrease in the viscosity of the solution with the increase in temperature, which leads to an increase in the rate of intra- and inter-particle diffusion of the dye molecules. The increase in the diffusion rate of dye molecules facilitates the movement of the dye to the adsorbent surface, thus increasing the adsorption capacity with the increase in solution temperature.

The thermodynamic parameters at different temperature are shown in Table 5, it shows that ΔG^0^ is less than zero for all five dyes during adsorption, indicating that the adsorption process is thermodynamically feasible and spontaneous. The values of |ΔG^0^| are all less than 20.00 kJ mol^−1^, demonstrating that the adsorption of these dyes on ARCNF is primarily by physical adsorption [23]. A positive value of ΔH^0^ further indicates that the adsorption process of ARCNF on the five dyes is endothermic reaction. Meanwhile, a positive value of ΔH^0^ suggests an increase in the disorder of the solid solution system and a strong affinity of the ARCNF for all five dyes, suggesting a considerable interaction between the active sites of ARCNF and the dye molecules [52].

#### 3.2.4. The Influence of pH on Adsorption of Organic Dyes in ARCNF

**Figure 8:**
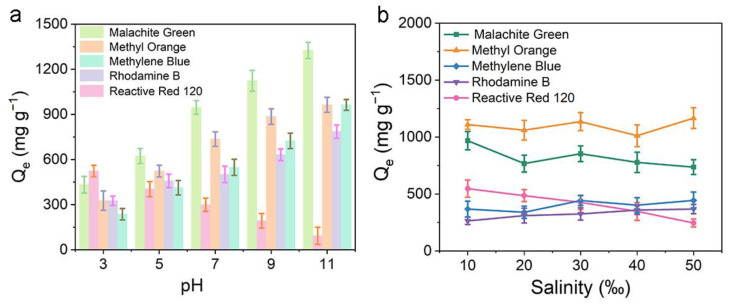
The influences of pH (**a**) and salinity (**b**) on adsorption of organic dyes in ARCNF.

The influence of pH on adsorption is a significant factor [13]. By changing the pH of the solution, the functional groups can be generated by the interaction between the adsorbent and the dye molecules [20]. The adsorption experiment was performed in a 20 mg L^−1^ dye solution with a pH range from 3 to 11, as shown in Figure 8a. The maximum adsorption of RR-120 was observed at pH = 3, and the adsorption of RR-120 by ARCNF gradually decreased with increasing pH to 11. At lower pH, RR-120 was negatively charged because of its sulfonated groups. Based on the zeta potential, it is known that ARCNF is positively charged at pH < 4.78. The electrostatic interaction between the negatively charged RR-120 and the positively charged ARCNF leads to the highest adsorption under acidic conditions. For the other four cationic dyes, the adsorption capacity gradually increased with increasing pH, suggesting that electrostatic forces play a dominant role in the adsorption process. When the pH is low, a large amount of H^+^ is present in the solution, and the excess H^+^ competes with these dye ions for the limited active sites on the ARCNF surface, thereby hindering their adsorption. As the pH increases, the H^+^ concentration in solution reduces and the OH^−^ concentration rises. The exclusion between the four dye ions and H^+^ decreases, thus enhancing their adsorption.

#### 3.2.5. The Influence of Salinity on Adsorption of Organic Dyes in ARCNF

In order to simulate the salinity variation of organic dyes in real wastewater, the effect of ionic strength on ARCNF adsorption during the adsorption process was explored by setting different salinity gradient (10–50‰) [7]. As shown in Figure 8b, there was basically no significant change in the adsorption of the four cationic dyes by the ARCNF during the gradual increase of ionic strength, further demonstrating the ability of ARCNF is able to maintain a high adsorption capacity in different ionic strength environments. It is remarkable that the adsorption of RR-120 gradually decreases during the increasing ionic strength, illustrating that salinity has an effect on its adsorption and electrostatic adsorption plays a dominant role in this process.

#### 3.2.6. Adsorption Mechanism

**Figure 9:**
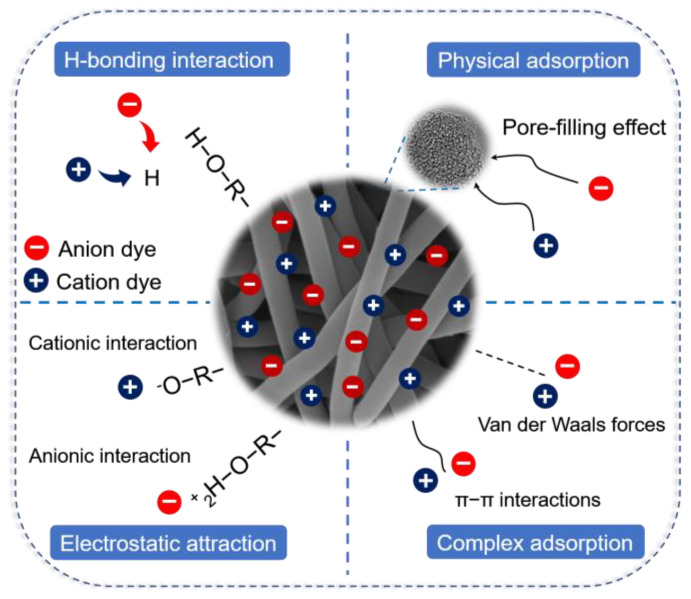
The possible adsorption mechanism of ARCNF with organic dyes.

From the above characterization and analysis of adsorption experiments, it can be concluded that the adsorption mechanism of the five organic dyes on the surface of ARCNF is determined by both physical and chemical adsorption, as shown in Figure 9. The abundant oxygen-containing functional groups (e.g., C−O, C=O, and O−C=O) on the surface of ARCNF can generate strong interactions with the dye molecules through hydrogen bonds, van der Waals forces and π−π interactions [41]. ARCNF with large specific surface area and rich pore structure provides a large number of active sites and ion transport channels for dye adsorption, which improves the adsorption efficiency [21]. Electrostatic attraction can be regarded as one of the most critical forces between the adsorbent and the organic dyes. Therefore, organic dyes can also be adsorbed through physical methods such as pore diffusion and surface adsorption.

#### 3.2.7. Regeneration Performance

**Figure 10:**
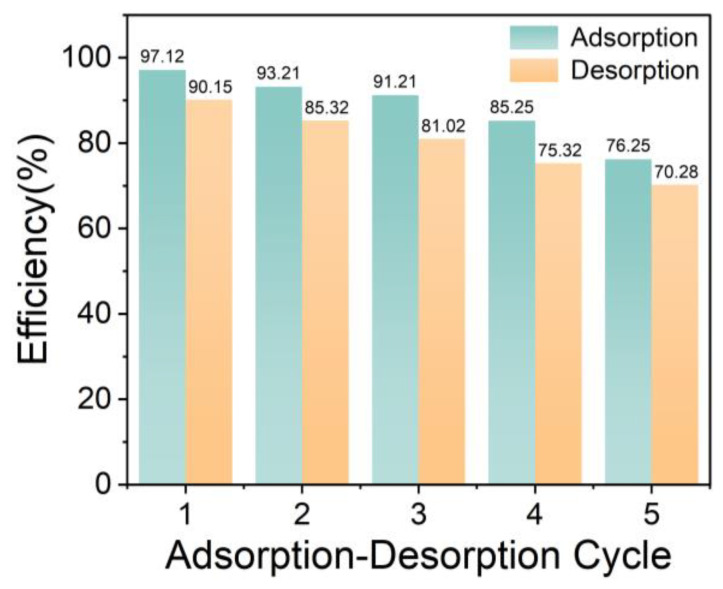
Reusability cycle performance of ARCNF for MG dye adsorption.

Regeneration of the adsorbent is very critical for its commercial application value [6]. In order to study the reusability of ARCNF, it was subjected to five adsorption/desorption cycles using ethanol/acetate acid eluate. MG with a higher adsorption capacity was selected as a typical dye for the cyclic experiment. From Figure 10, the adsorption capacity is still higher than 85% after 4 cycles, although it decreases with the increasing number of cycles. During desorption, part of the adsorption sites of ARCNF may be lost or blocked, thus reducing its adsorption capacity of MG dye after desorption. As a result, ARCNF exhibits excellent adsorption capacity even after several cycles and has the potential to be a reusable dye adsorbent.

## 4. Conclusions

In this work, we prepared ARCNF by introducing OCLR into the spinning solution containing PAN through electrostatic spinning technology. The ARCNF has enhanced mechanical properties with a Young’s modulus five times that of ACNF. It exhibits large specific surface area (2504 m^2^ g^−1^) and high pore volume (1.25 cm^3^ g^−1^). ARCNF demonstrates excellent adsorption capacity on cationic dyes (MG, MO, MB, and RB) and anionic dye (RR-120). The adsorption of these dyes in ARCNF are consistent with the PSOKM and Langmuir isotherm model. The ARCNF has a maximum adsorption capacity of 2712.84 mg g^−1^ for MG, 2210.58 for MO, 1017.27 for MB, 867.21 for RB, and 719.49 for RR-120. In addition, the thermodynamic studies indicate that the adsorption of five organic dyes on ARCNF are spontaneous and endothermic processes. More importantly, the adsorption capacity for MG dye was still higher than 76% after five adsorption-desorption cycles. In conclusion, ARCNF has good adsorption performances on common organic dyes, which has great application potential as a renewable adsorbent for the removal of organic dyes from wastewater treatment in the future. Meanwhile, it also provides a powerful idea for the high value-added utilization of solid waste.

## Figures and Tables

**Table 1:** Dye names and abbreviations.

Dye Type	Dye	Abbreviation
Cationic	Malachite Green	MG
	Methyl Orange	MO
	Methylene Blue	MB
	Rhodamine B	RB
Anionic	Reactive Red 120	RR-120

**Table 2:** Adsorption kinetics parameters of ARCNF for different organic dyes.

Dye	Pseudo-First Order					Pseudo-Second Order		
	*q_1_*	*K*_1_	R^2^	X^2^	*q_2_*	*K*_2_ × 10^−5^	R^2^	X^2^
MG	695.54	0.033	0.997	149.7	923.03	3.24	0.998	122.0
MO	594.98	0.036	0.997	111.5	789.43	3.97	0.998	72.29
MB	314.82	0.053	0.987	197.2	391.16	0.14	0.992	127.9
RB	298.08	0.046	0.993	84.99	382.31	0.14	0.995	59.16
RR-120	253.65	0.034	0.996	43.15	338.15	8.86	0.997	23.29

**Table 3:** Adsorption isotherm parameters of ARCNF for different organic dyes.

Dye	Langmuir Model				Freundlich Model			
	q_max_	K_L_	R^2^	X^2^	K_F_	n	R^2^	X^2^
MG	2712.84	0.015	0.986	1306.87	170.53	0.657	0.963	2058.18
MO	2210.58	0.012	0.975	880.38	116.55	0.685	0.962	1281.32
MB	1017.27	0.029	0.979	360.65	119.05	0.506	0.974	657.98
RB	867.21	0.028	0.989	542.26	96.85	0.517	0.958	745.16
RR-120	719.49	0.019	0.986	453.37	57.23	0.597	0.975	824.36

**Table 4:** Comparison of maximum adsorption capacity for ARCNF with various adsorbents.

Adsorbent	Dye	Adsorption Capacity (mg g^−1^)	Ref.
Activated carbon fiber	MO	357.14	[11]
Hyper-crosslinked polymers	MB	766.74	[13]
Shaddock peel activated carbon	MB	859.81	[20]
Freeze-dried carbon microsphere	RB	216.61	[41]
Cyanobacteria-based porous carbon	MB	667	[42]
Activated graphene	MG	791.27	[46]
Magnetic multi-walled carbon nanotubes	MG	442.2	[47]
Chitosan-coated Fe_3_O_4_ nanocomposites	MO	758	[49]
Moringa oleifera seed	RR-120	413.32	[50]
Nano-biosorbents of pine needles	MB	1193	[51]
ARCNF	MG	2712.84	This study
ARCNF	MO	2210.58	
ARCNF	MB	1017.27	
ARCNF	RB	867.21	
ARCNF	RR-120	719.49	

**Table 5:** Thermodynamic parameters of adsorption of ARCNF to different dyes.

Dyes	ΔG^0^ (KJ mol^−1^K^−1^)			ΔS^0^ (J mol^−1^)	ΔH^0^ (KJ mol^−1^)
	288 K	298 K	308 K		
MG	−3.74	−4.11	−4.49	37.41	7.03
MO	−2.78	−3.27	−3.76	49.05	11.34
MB	−2.34	−2.79	−3.24	44.89	10.58
RB	−8.11	−8.60	−9.09	49.05	6.01
RR-120	−3.43	−3.80	−4.18	37.24	7.29

## Data Availability

Data sharing is not applicable to this article.

## Supplementary Materials

The following supporting information can be downloaded at: https://www.mdpi.com/article/10.3390/ma16103614/s1, Figure S1: Diameter distribution of ACNF. Figure S2: HRTEM images of OCLR. Figure S3: Confidence intervals for quasi-secondary adsorption kinetics of different organic dyes. Figure S4: Adsorption isotherm of different dyes on ARCNF: (a, c, e, g, i) concentration, (b, d, f, h, j) Freundlich and Langmuir isotherm fitting; Figure S5: Adsorption thermodynamics of different organic dyes on ARCNF. Figure S6: Adsorption isotherms and isotherm fitting of different dyes in ARCNF at different temperatures; Table S1: Texture properties of the ARCNF measured by N_2_ adsorption-desorption isotherms; Table S2: The XPS survey spectra and relative contents of the components from XPS C 1s in ARCNF.
