# Supplementary material for: Preparation of Reusable Porous Carbon Nanofibers from Oxidized Coal Liquefaction Residue for Efficient Adsorption in Water Treatment
Source: Materials (Basel). 2023 May 9;16(10):3614. doi: 10.3390/ma16103614 (PMC10222528; doi:10.3390/ma16103614)
Supplement: Supplementary file 1 [file materials-16-03614-s001.zip › materials-2348005-supplementary.pdf]

materials

Supplementary Information

Preparation of Reusable Porous Carbon Nanofibers from Oxidized Coal Liquefaction Residue for Efficient Adsorption in Water Treatment

Yaoyao Chen [1], Kefu Wang [1], Liqin Cao [1], Xueli Huang [1,*] and Yizhao Li [1,2,*]

[1] State Key Laboratory of Chemistry and Utilization of Carbon Based Energy Resources, College of Chemical Engineering and Technology, Xinjiang University, Urumqi 830017, China; cey0822@163.com (Y.C.); awkf0924@163.com (K.W.); cao_lq@xju.edu.cn (L.C.)
[2] Yangtze Delta Region Institute (Huzhou), University of Electronic Science and Technology of China, Huzhou 313001, China
* Correspondence: xuelih@163.com (X.H.); liyizhao0809@126.com (Y.L.); Tel.: +86-991-8583083 (Y.L.); Fax: +86-991-8588883 (Y.L.)

Citation: Chen, Y.; Wang, K.; Cao, L.; Huang, X.; Li, Y. Preparation of Reusable Porous Carbon Nanofibers from Oxidized Coal Liquefaction Residue for Efficient Adsorption in Water Treatment. *Materials* **2023**, *16*, 3614. https://doi.org/10.3390/ma16103614

Academic Editor: Roberta G. Toro

Received: 31 March 2023
Revised: 2 May 2023
Accepted: 5 May 2023
Published: 9 May 2023

Copyright: © 2023 by the authors. Licensee MDPI, Basel, Switzerland. This article is an open access article distributed under the terms and conditions of the Creative Commons Attribution (CC BY) license (https://creativecommons.org/licenses/by/4.0/).

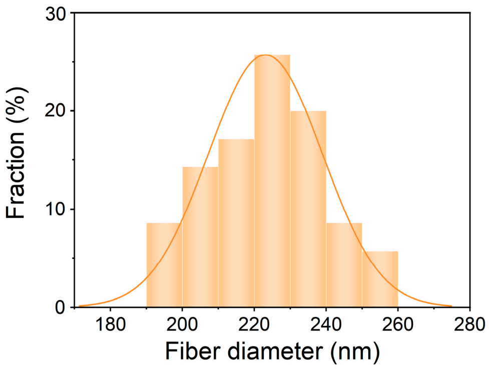

Figure S1. Diameter distribution of ACNF.

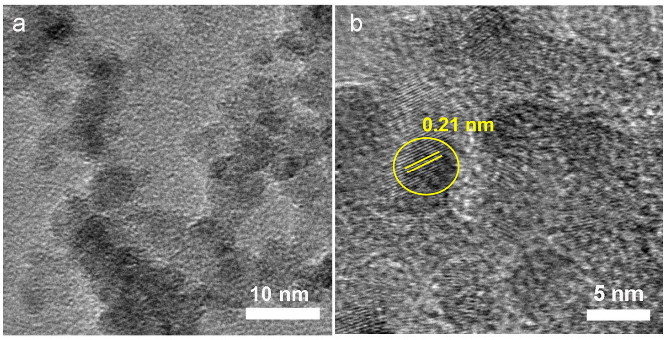

Figure S2. HRTEM images of OCLR.

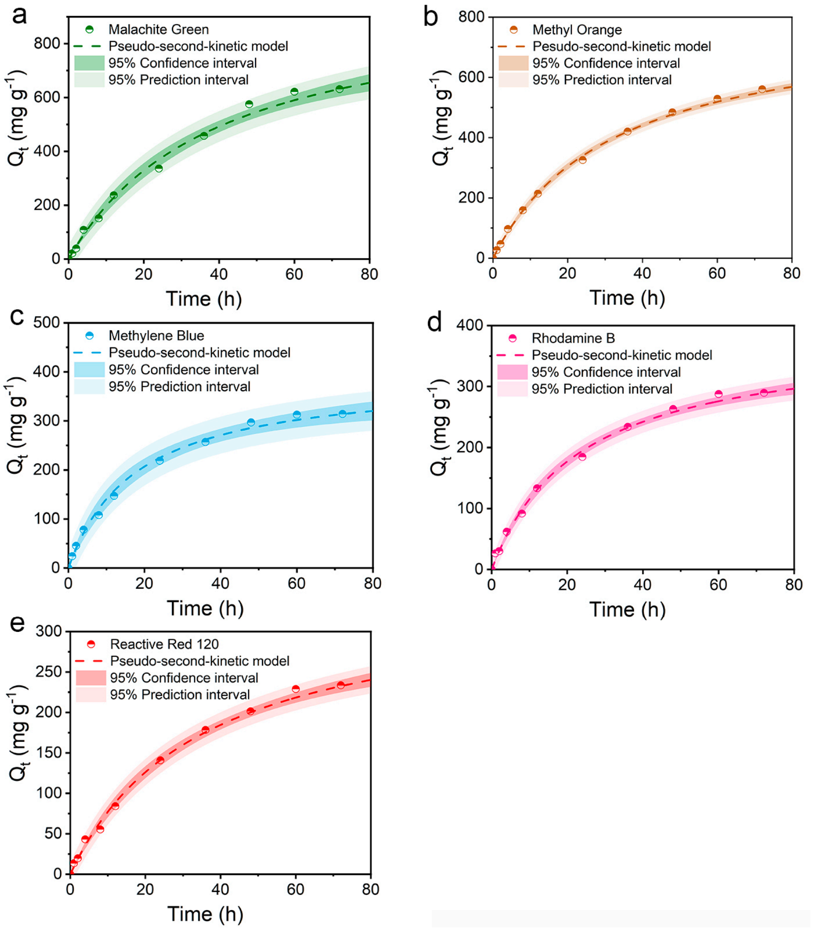

Figure S3. Confidence intervals for quasi-secondary adsorption kinetics of different organic dyes.

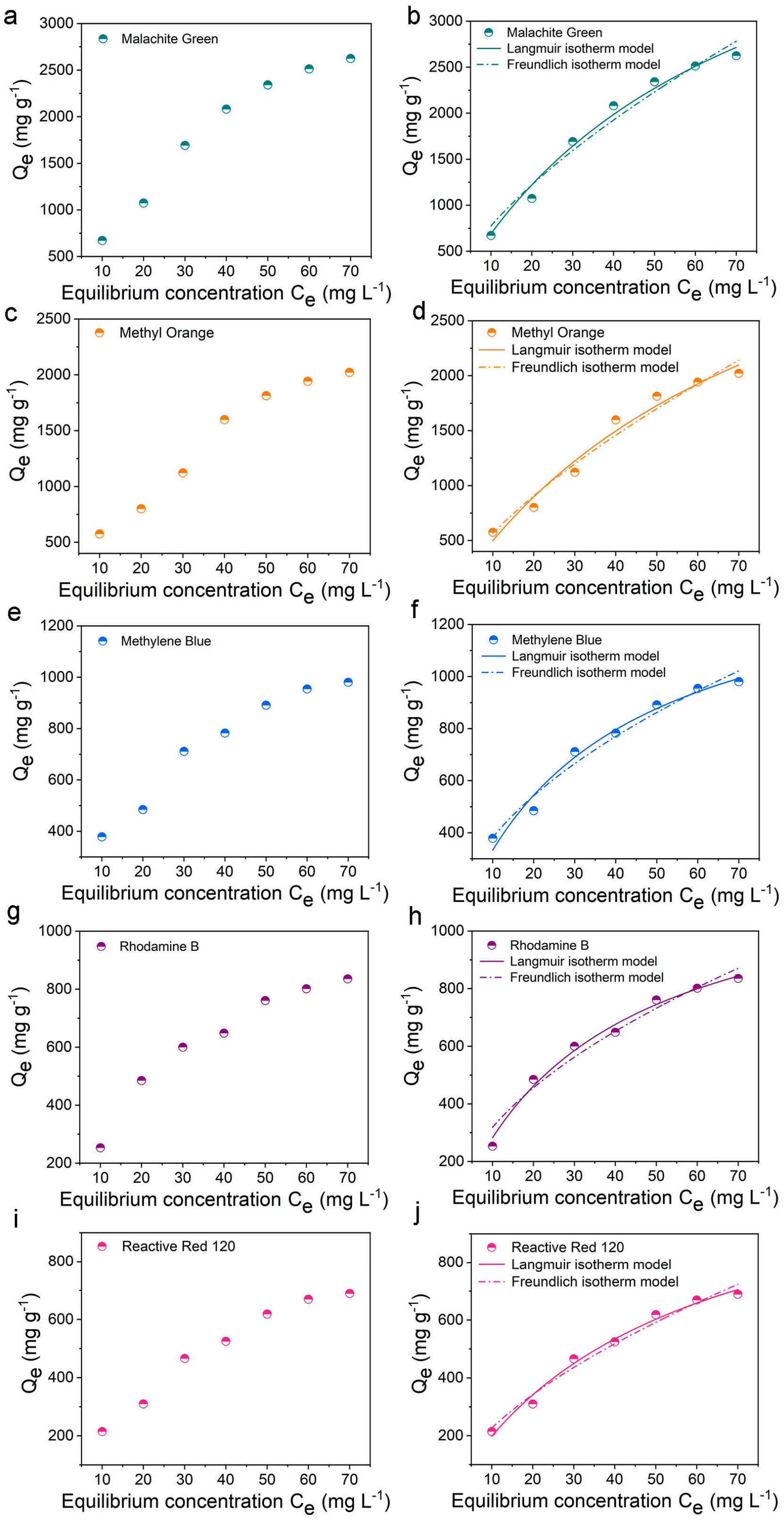

Figure S4. Adsorption isotherm of different dyes on ARCNF: (a, c, e, g, i) concentration, (b, d, f, h, j) Freundlich and Langmuir isotherm fitting.

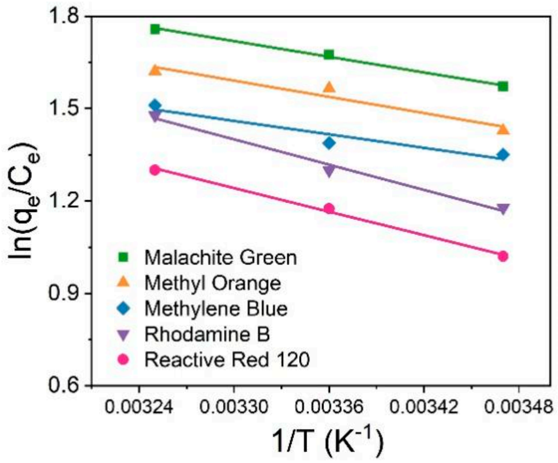

Figure S5. Adsorption thermodynamics of different organic dyes on ARCNF.

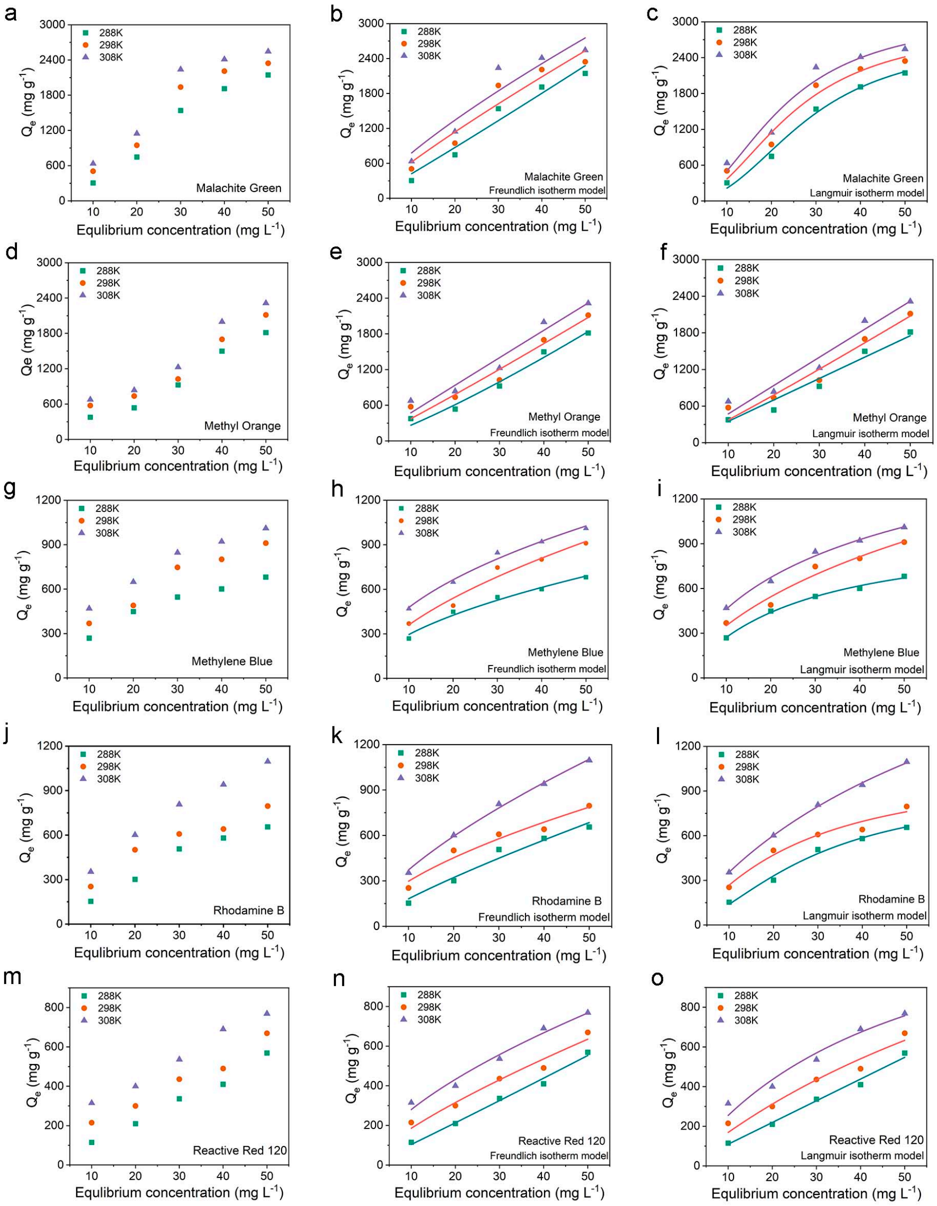

Figure S6. Adsorption isotherms and isotherm fitting of different dyes in ARCNF at different temperatures.

Table S1. Texture properties of the ARCNF measured by N_2 adsorption-desorption isotherms.

Sample	S_{BET} a) (m^2 g^{-1})	V_{total} b) (cm^3 g^{-1})	V_{micro} c) (cm^3 g^{-1})	V_{mecro} d) (cm^3 g^{-1})	V_{micr}/ V_{mecro}
ARCNF	2504	1.25	0.24	1.01	0.24

[a] Specific surface area calculated by BET method.

[b] Total pore volume.

[c] Volume of micropores.

[d] Volume of mesopores.

Table S2. The XPS survey spectra and relative contents of the components from XPS C 1s in ARCNF.

Sample	Element composition (at. %)			Contribution of the components in C 1s spectra (at. %)		
	C	O	N	C=C	C–O/C-N	C=O
ARCNF	88.1	9.7	2.2	62	24	14
